# Supplementary figures and images for: Local-scale impact of wind energy farms on rare, endemic, and threatened plant species
Source: PeerJ. 2021 May 19;9:e11390. doi: 10.7717/peerj.11390 (PMC8140595; doi:10.7717/peerj.11390)

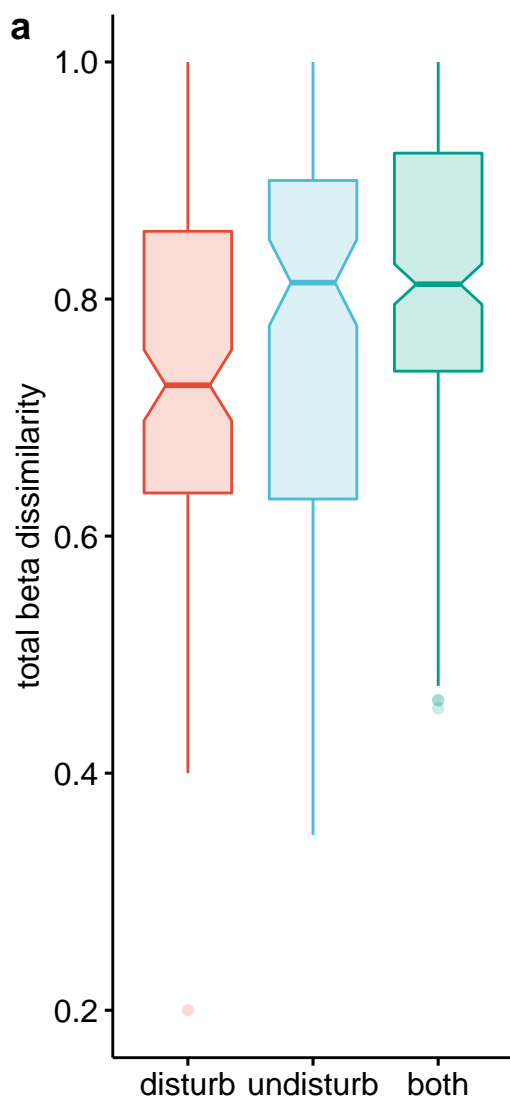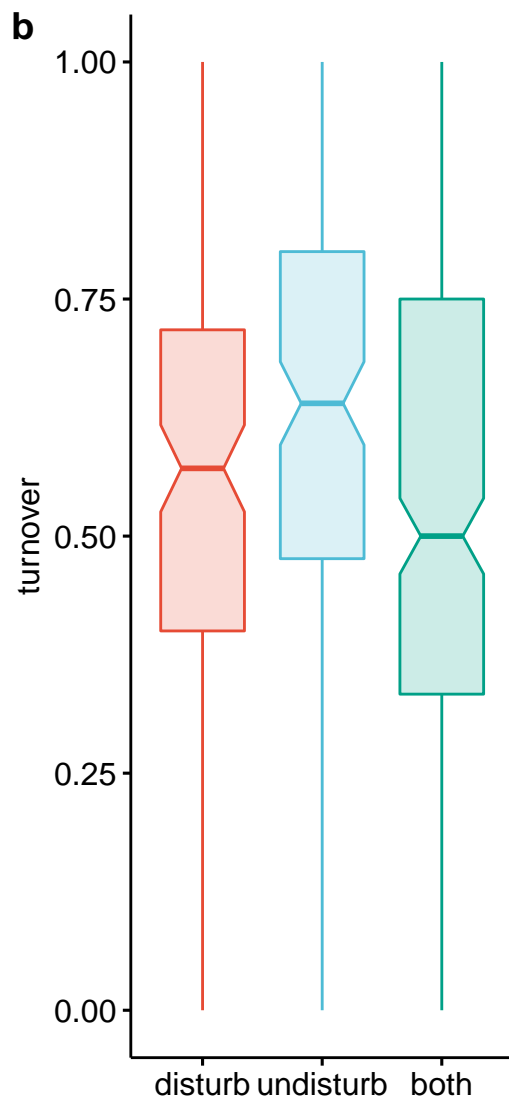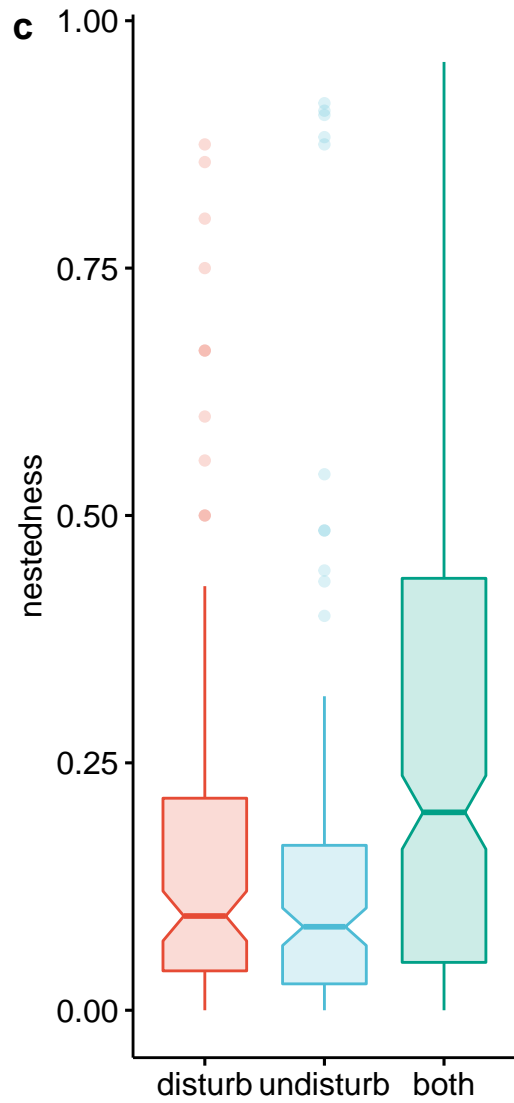

Supplement: Supplemental Information 2 [file peerj-09-11390-s002.zip › R_code_windt_impact/Output/beta.diversity.graph.pdf]

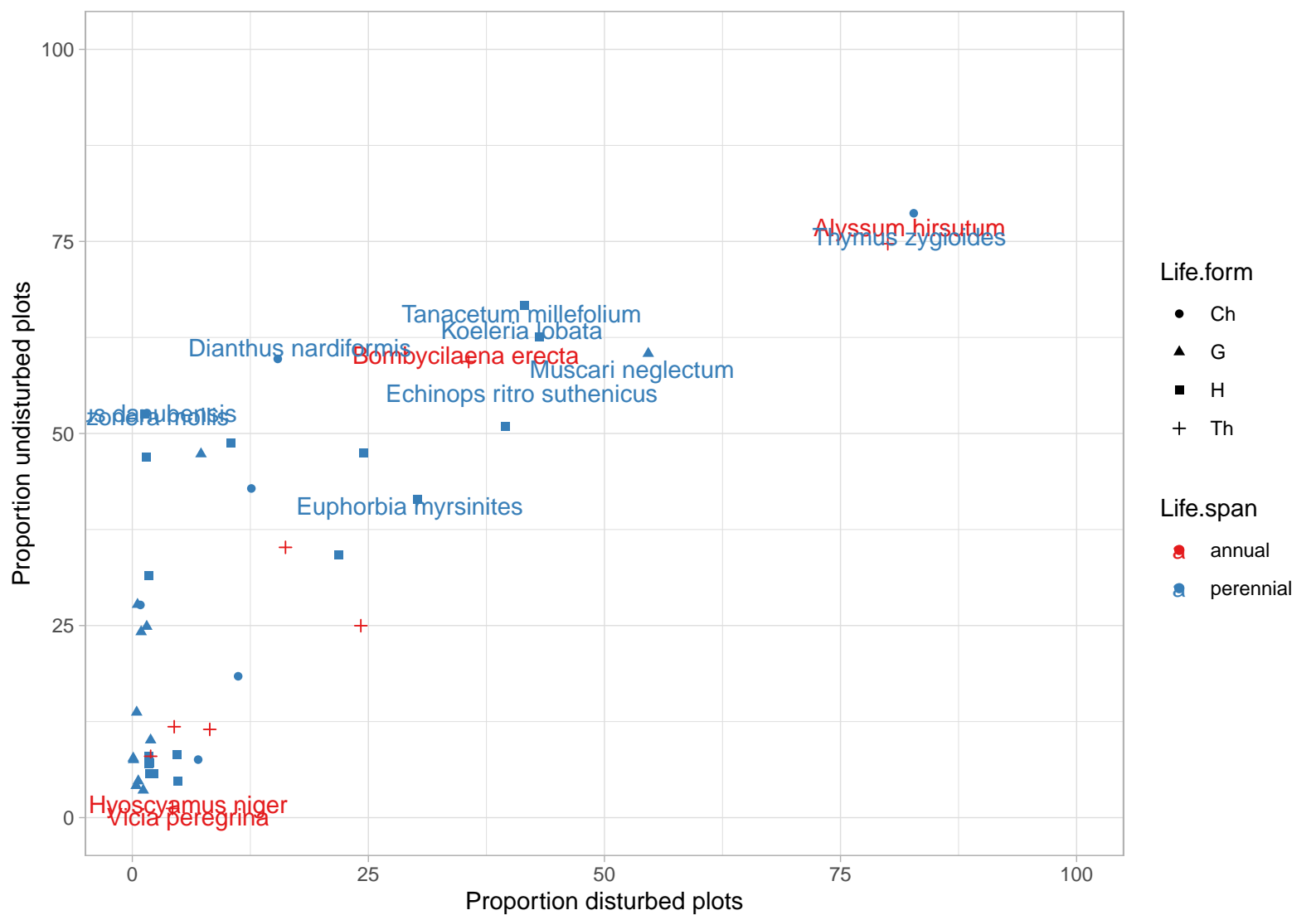

Supplement: Supplemental Information 2 [file peerj-09-11390-s002.zip › R_code_windt_impact/Output/frequency.pdf]

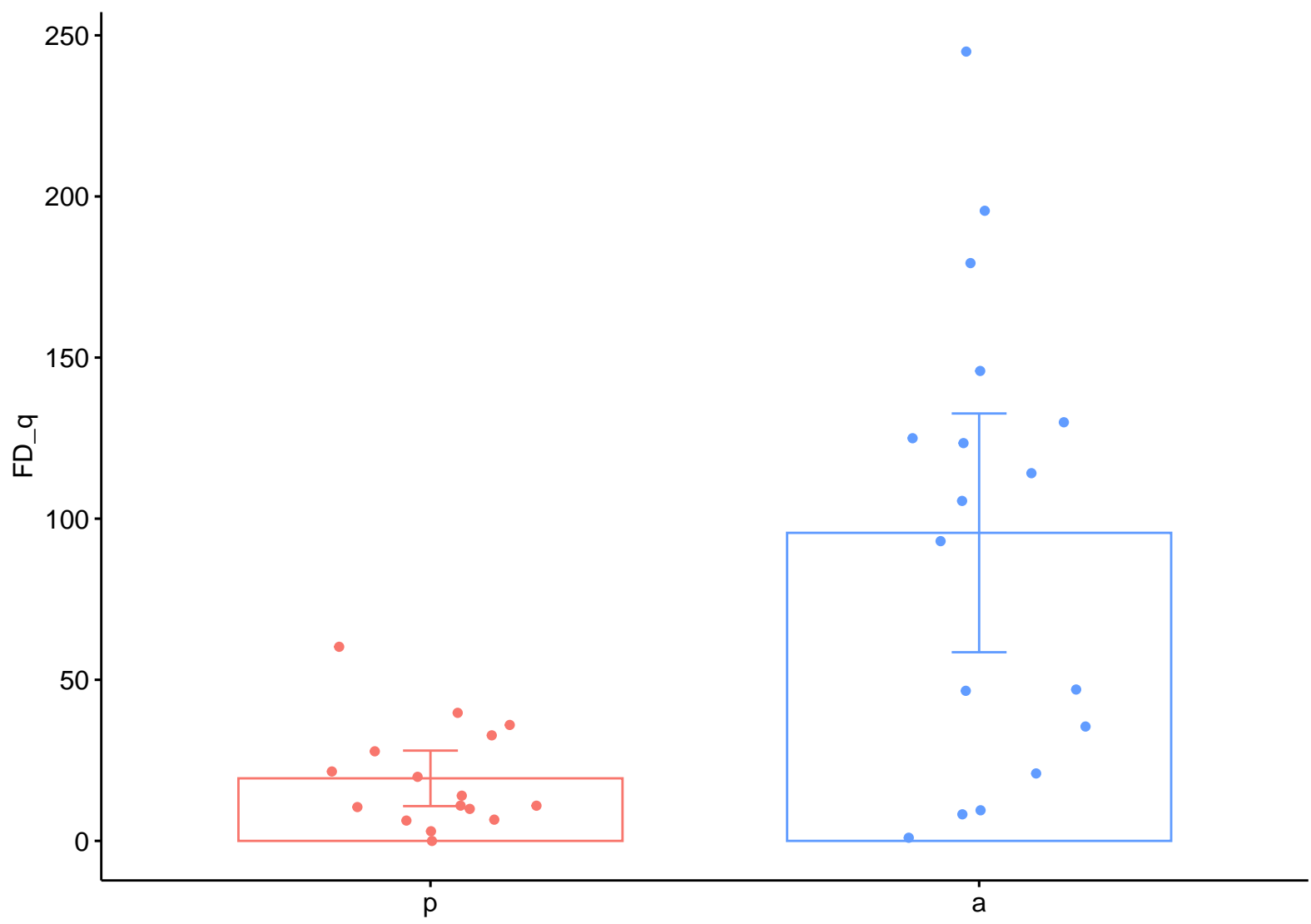

Supplement: Supplemental Information 2 [file peerj-09-11390-s002.zip › R_code_windt_impact/Output/functional_diversity.pdf]

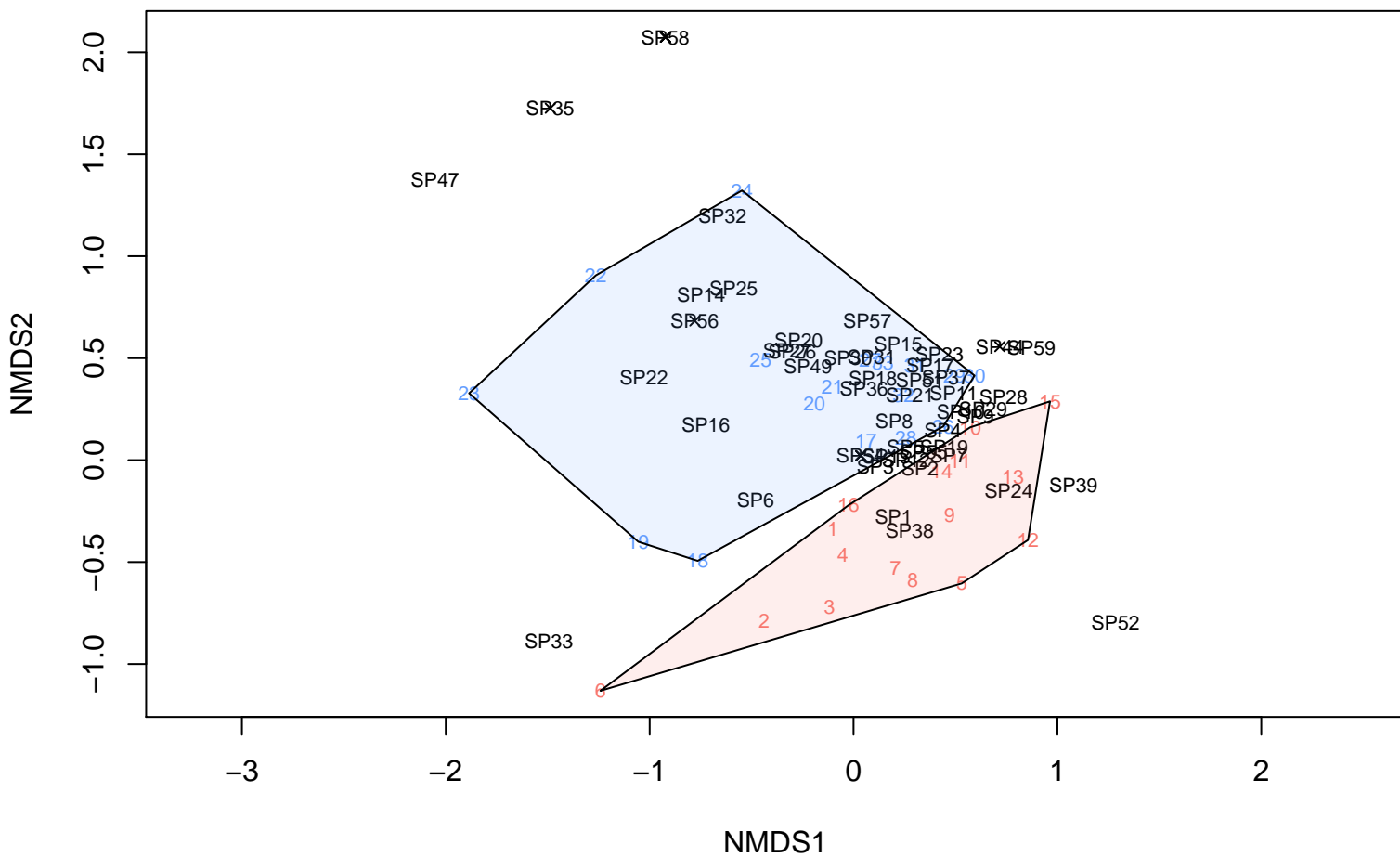

Supplement: Supplemental Information 2 [file peerj-09-11390-s002.zip › R_code_windt_impact/Output/NMDS.pdf]

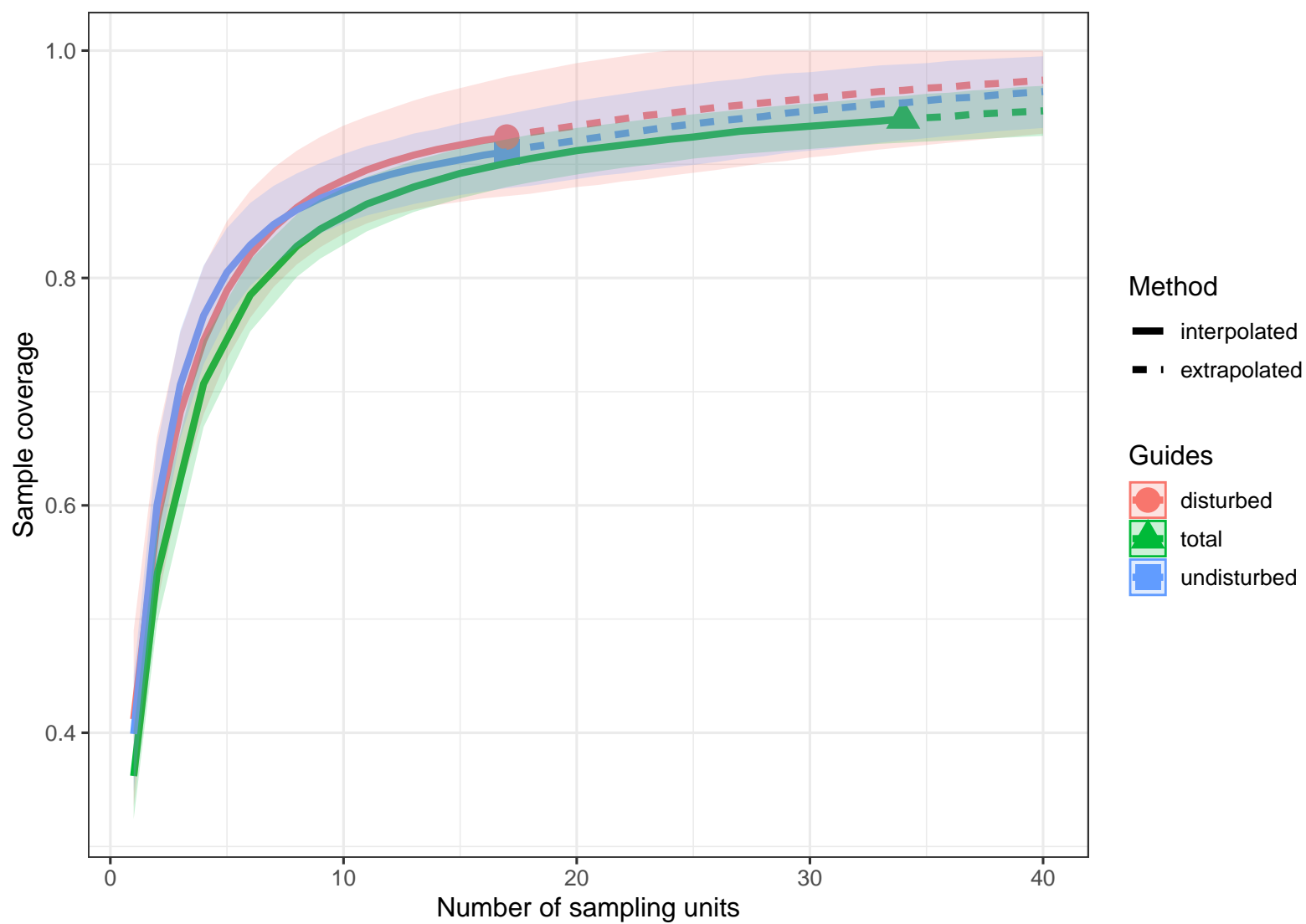

Supplement: Supplemental Information 2 [file peerj-09-11390-s002.zip › R_code_windt_impact/Output/RE_sample_completeness.pdf]

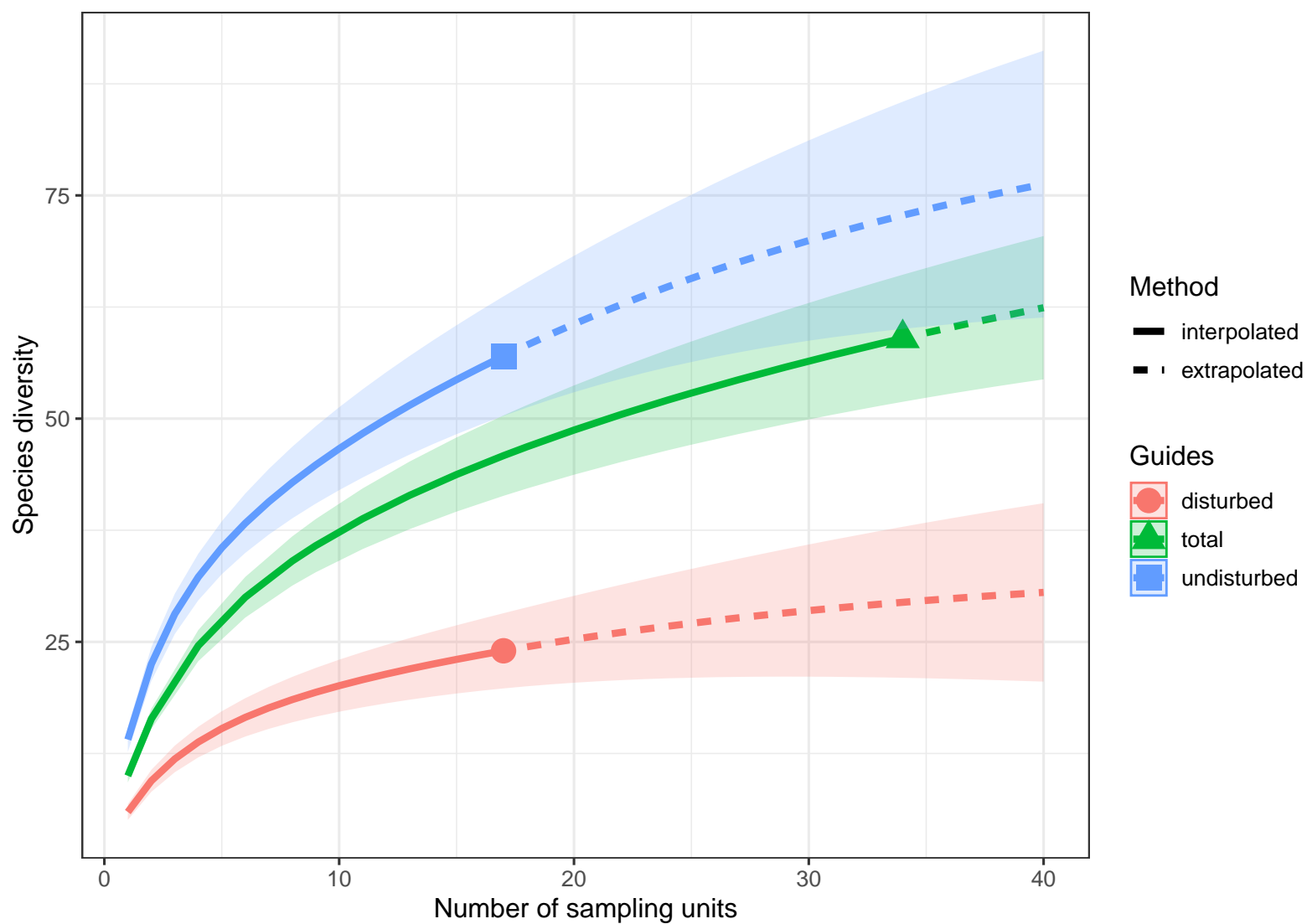

Supplement: Supplemental Information 2 [file peerj-09-11390-s002.zip › R_code_windt_impact/Output/RE_sample_size.pdf]

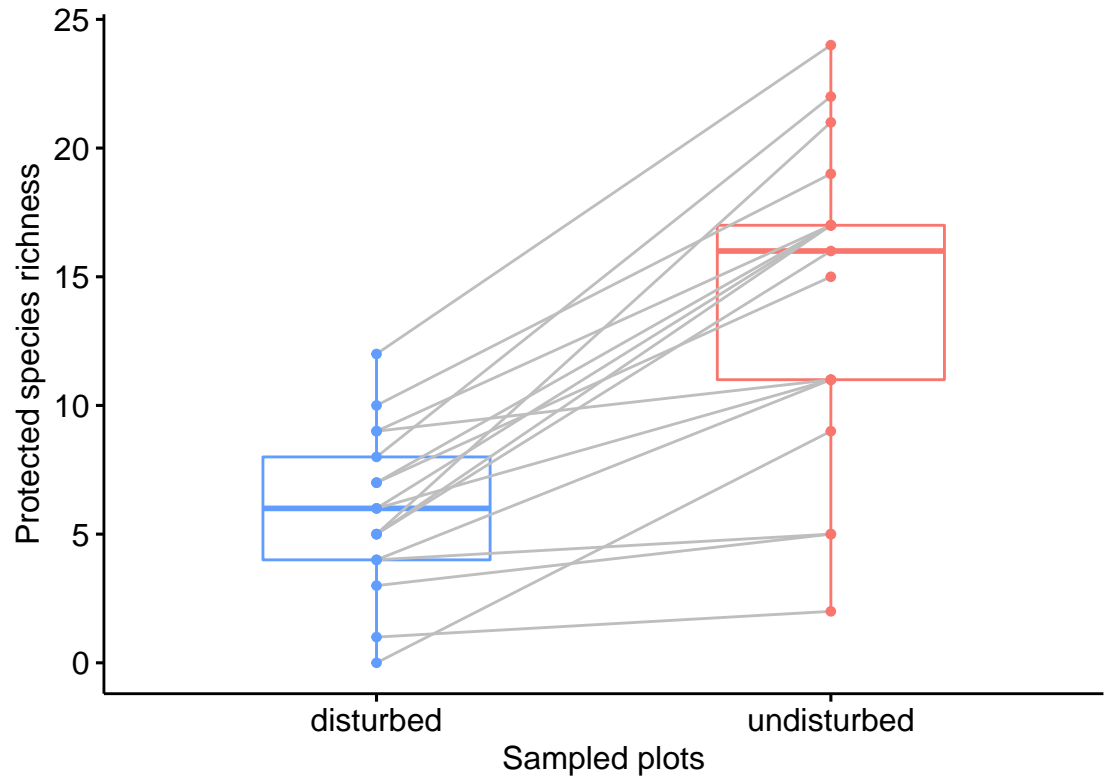

Supplement: Supplemental Information 2 [file peerj-09-11390-s002.zip › R_code_windt_impact/Output/richness.pair.pdf]

Ordination Distance

Non-metric fit,  $R^2 = 0.97$   
Linear fit,  $R^2 = 0.882$

Observed Dissimilarity

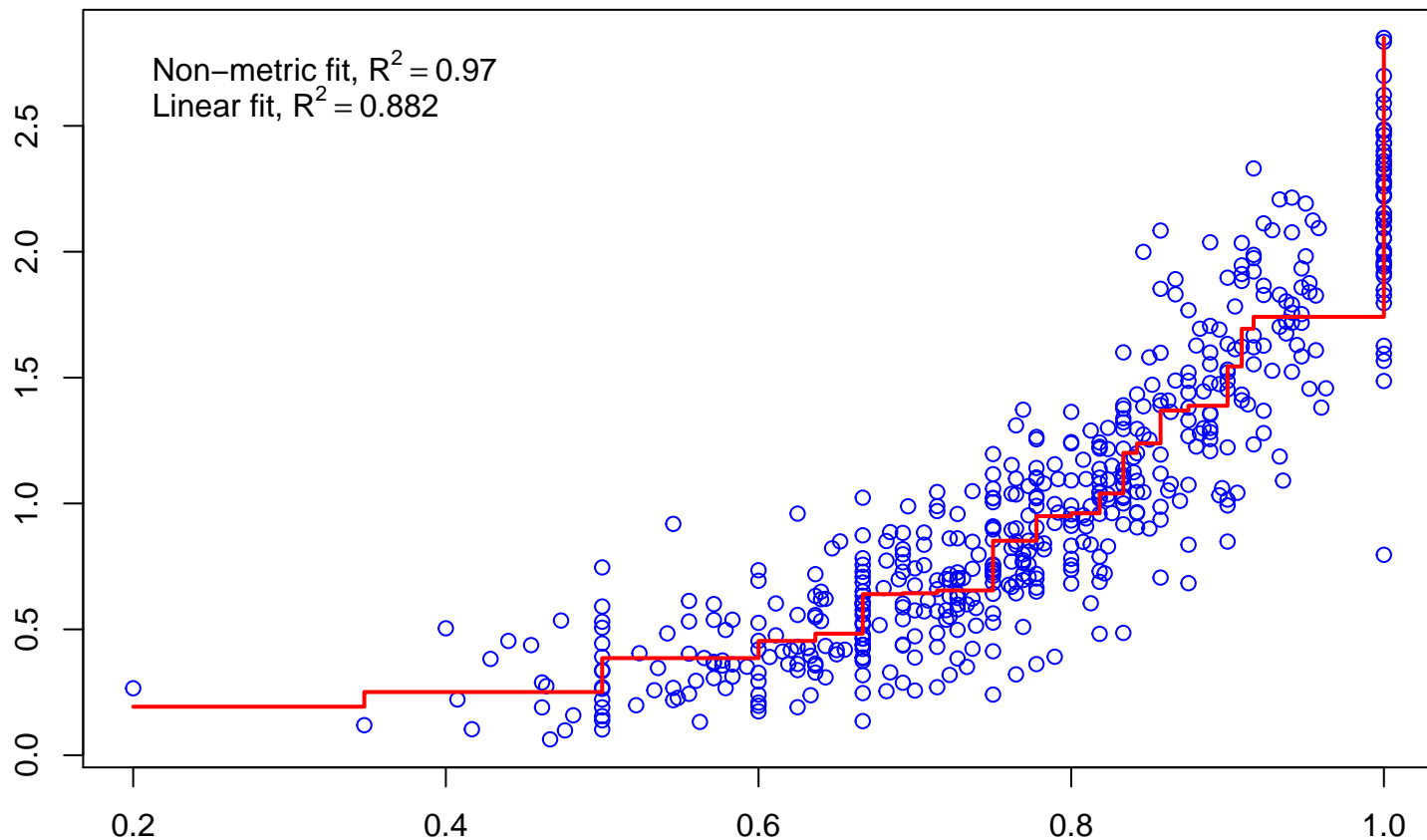

Supplement: Supplemental Information 2 [file peerj-09-11390-s002.zip › R_code_windt_impact/Output/Shepard_plot_nmds.pdf]

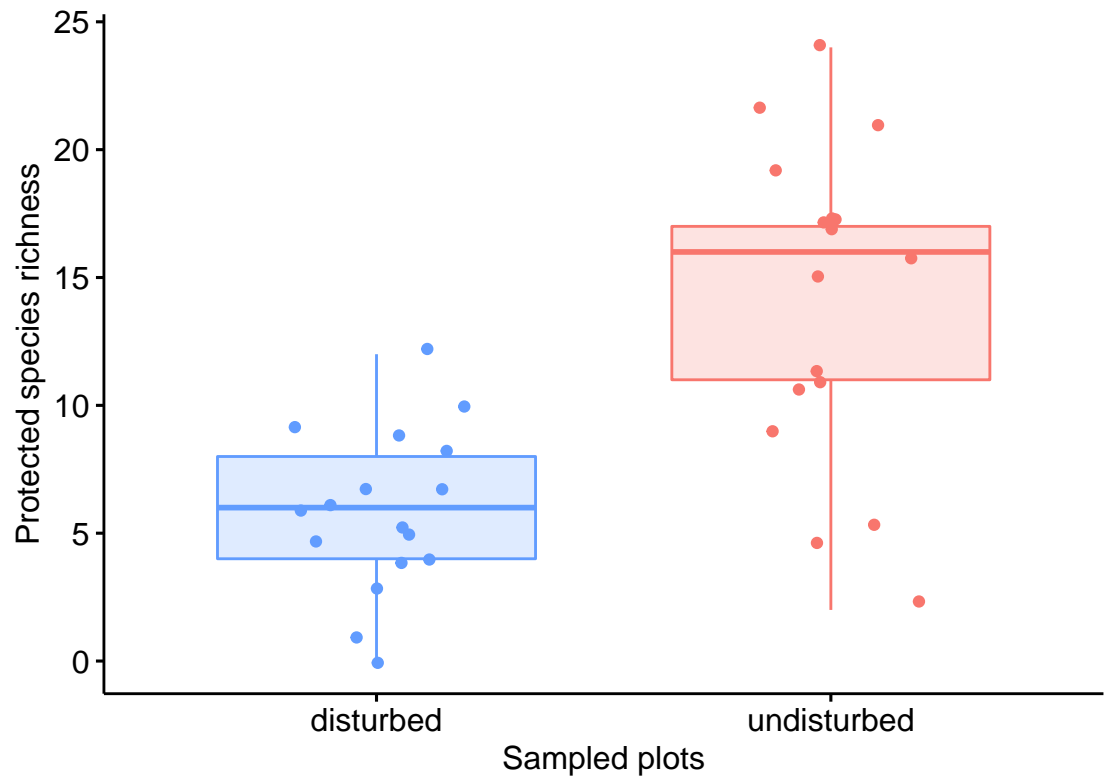

Supplement: Supplemental Information 2 [file peerj-09-11390-s002.zip › R_code_windt_impact/Output/total_richness.pdf]

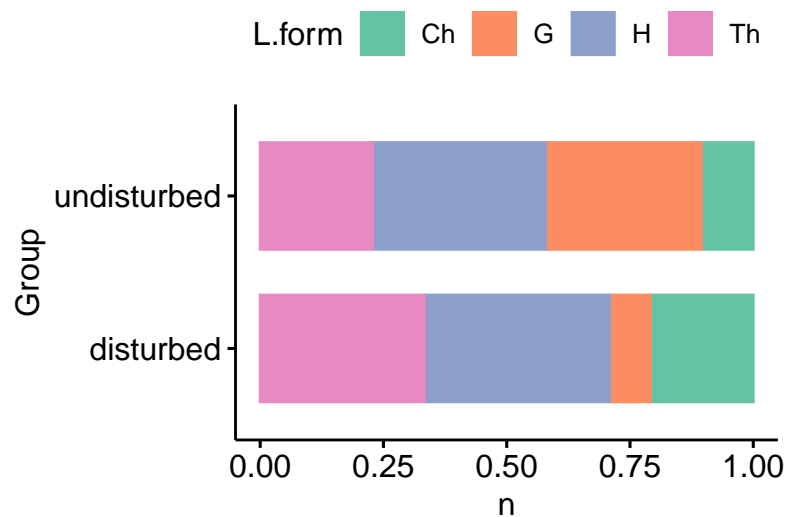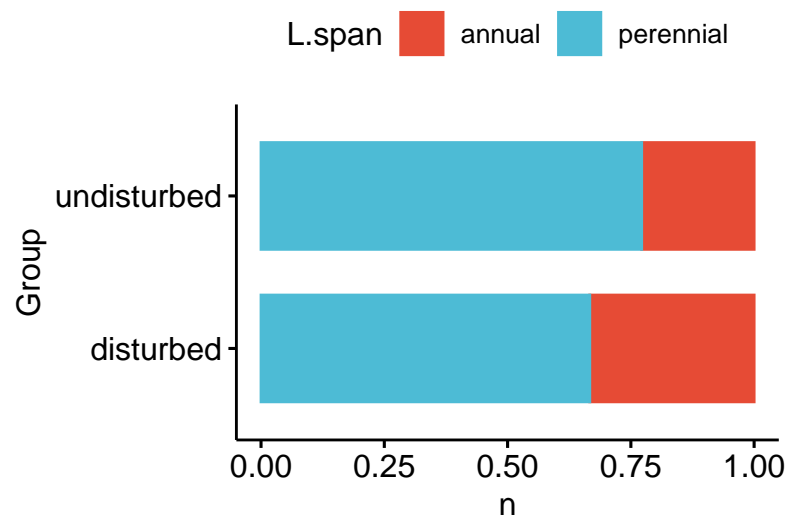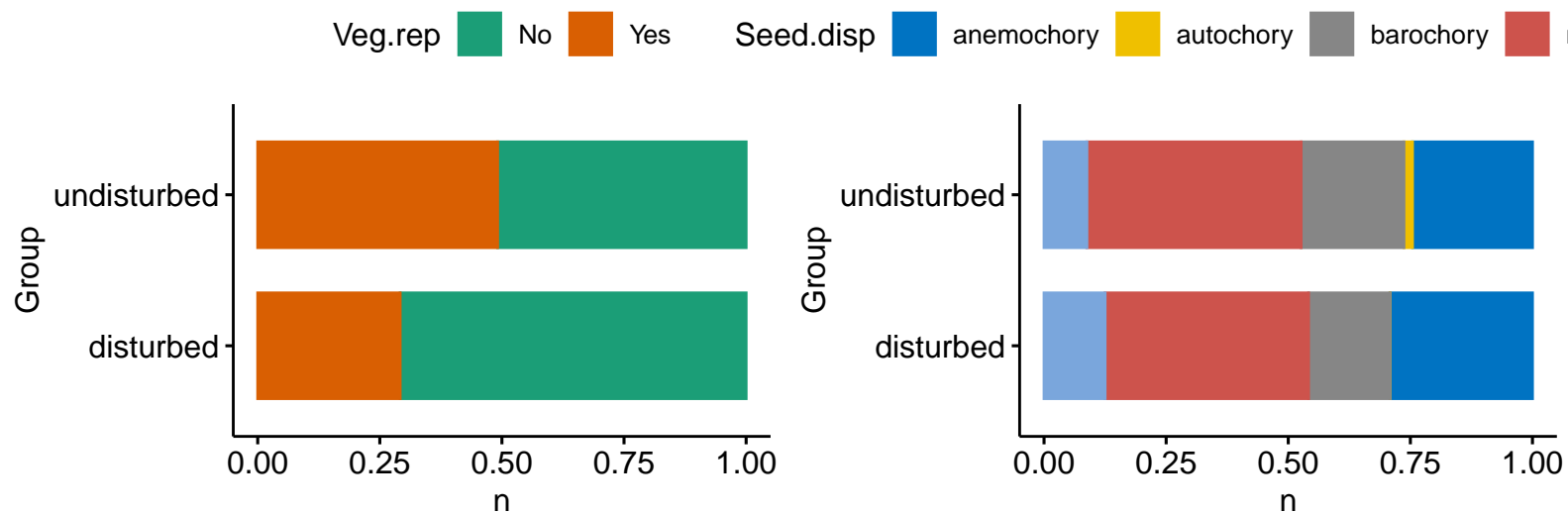

Supplement: Supplemental Information 2 [file peerj-09-11390-s002.zip › R_code_windt_impact/Output/traits.proportions.pdf]

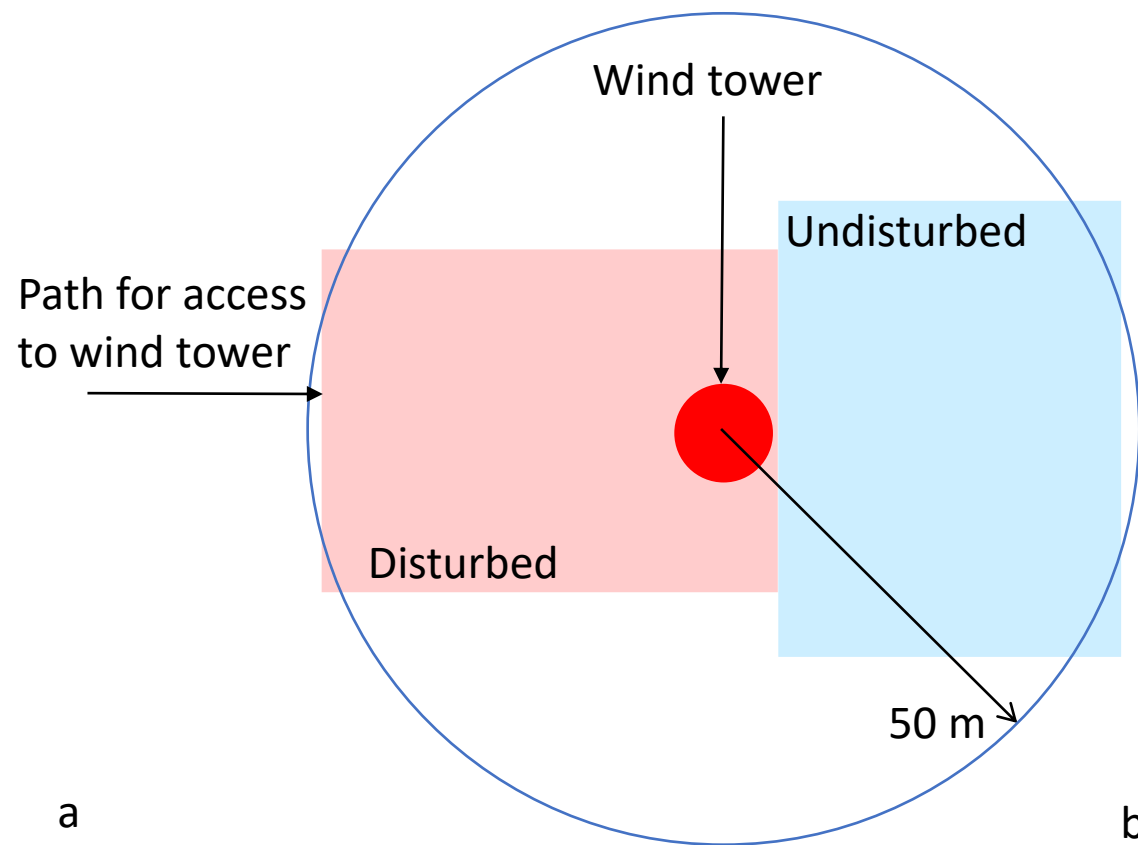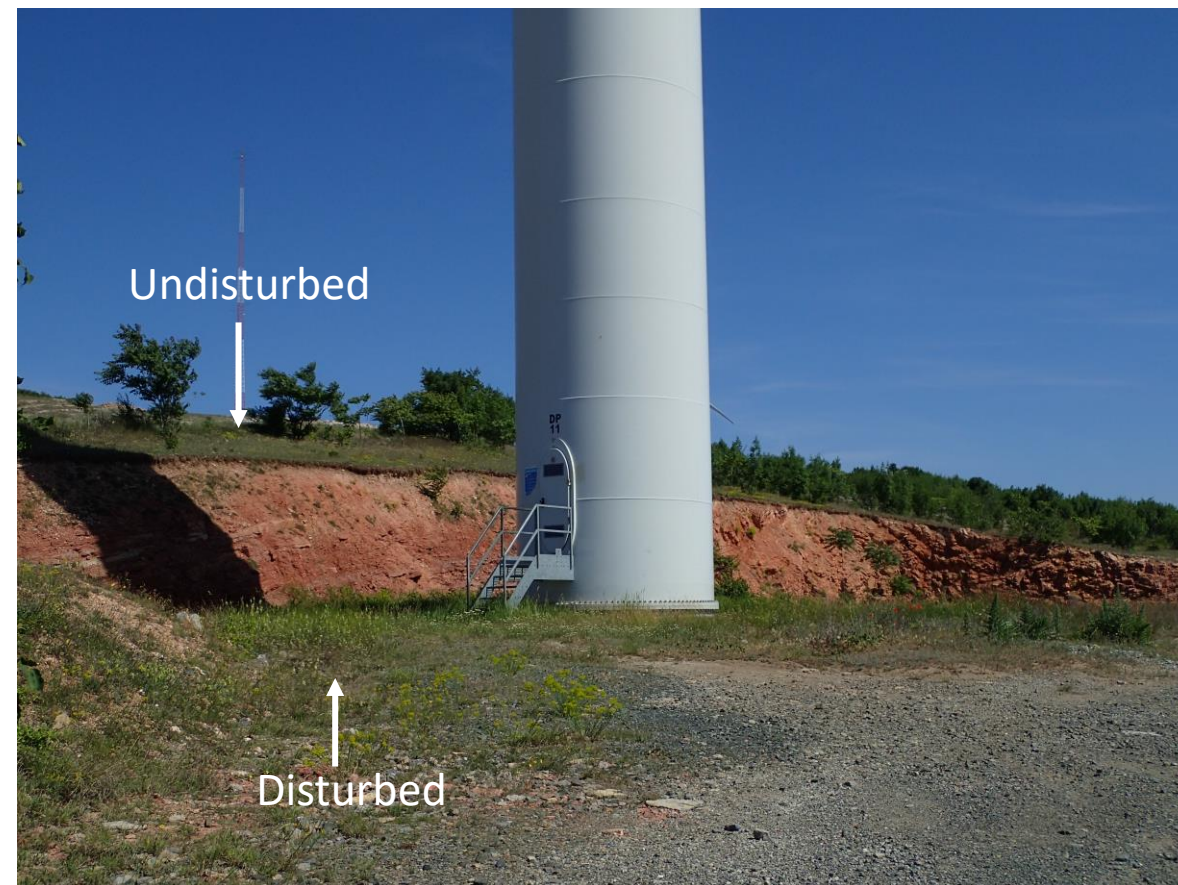

Supplement: Supplemental Information 3 — (A) The disturbed plot overlaps the technological platform of a wind tower, while the undisturbed plot is adjacent to the disturbed one, covering an area where vegetation was not directly impacted by the construction or operation of the wind farm. (B) Aspect with a wind tower (DP_11). In the first plan is the disturbed area; in the background is the undisturbed area. [file peerj-09-11390-s003.pdf]

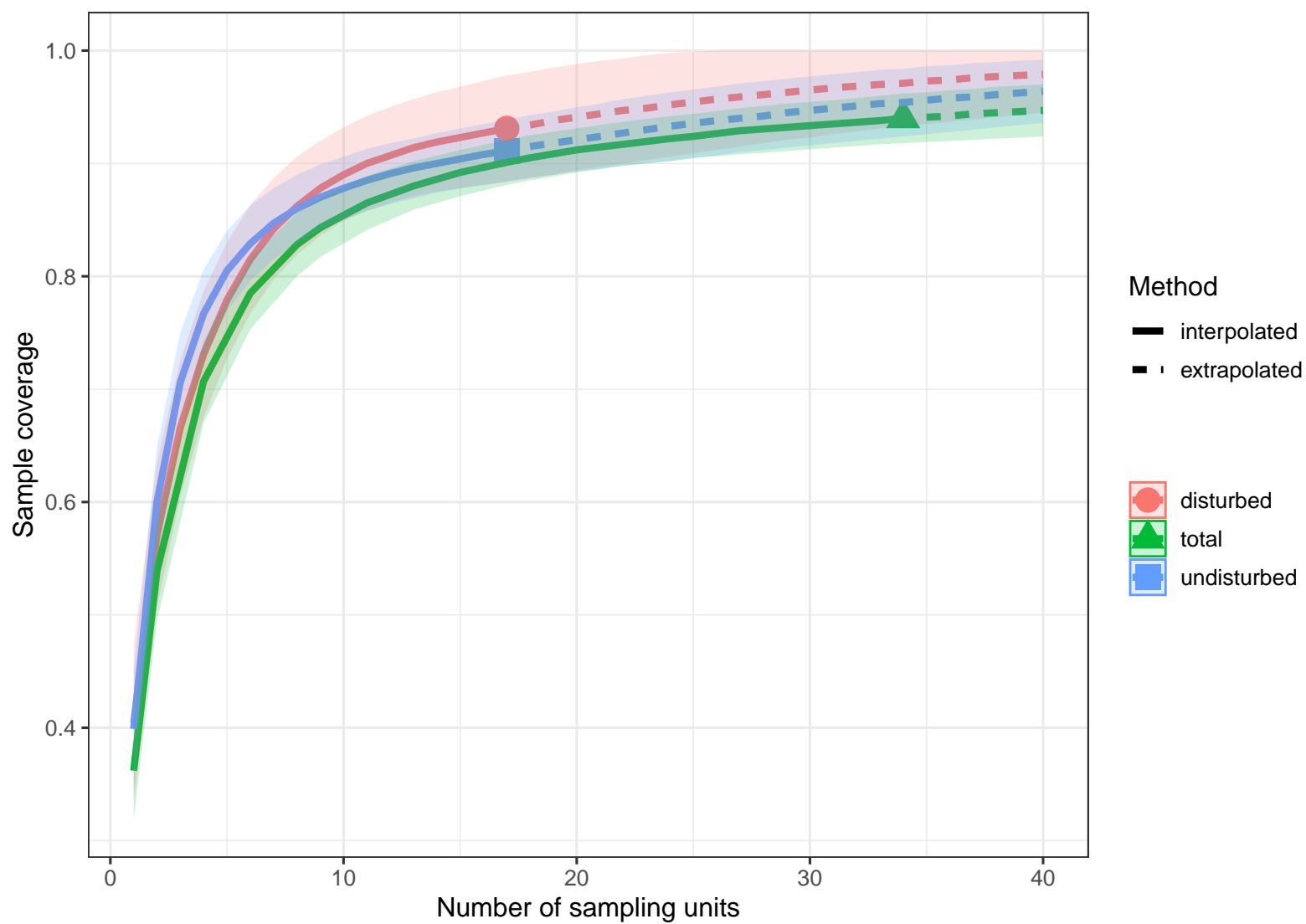

Supplement: Supplemental Information 4 [file peerj-09-11390-s004.pdf]

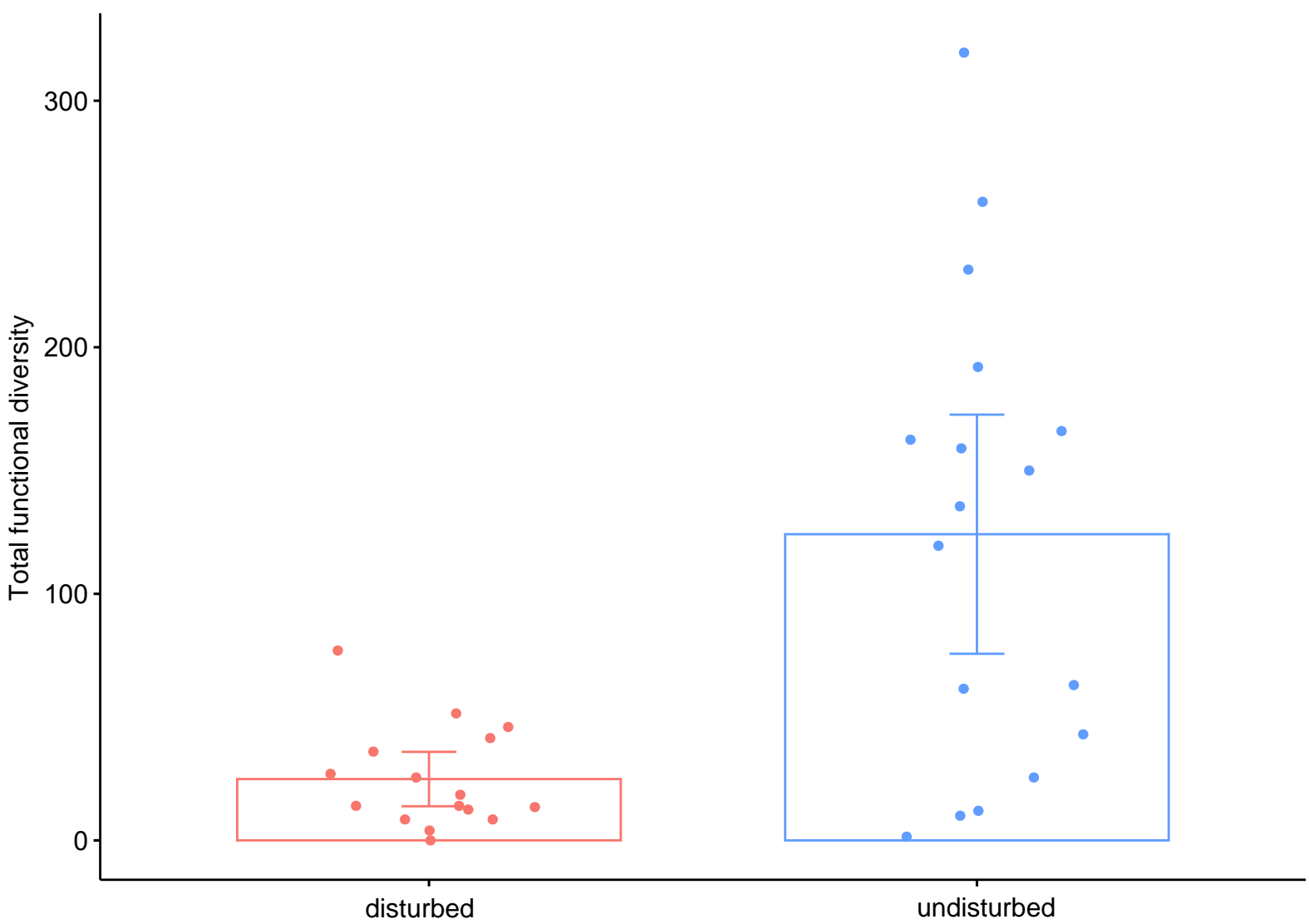

Supplement: Supplemental Information 5 [file peerj-09-11390-s005.pdf]

Ordination Distance

Non-metric fit,  $R^2 = 0.97$

Linear fit,  $R^2 = 0.882$

Observed Dissimilarity

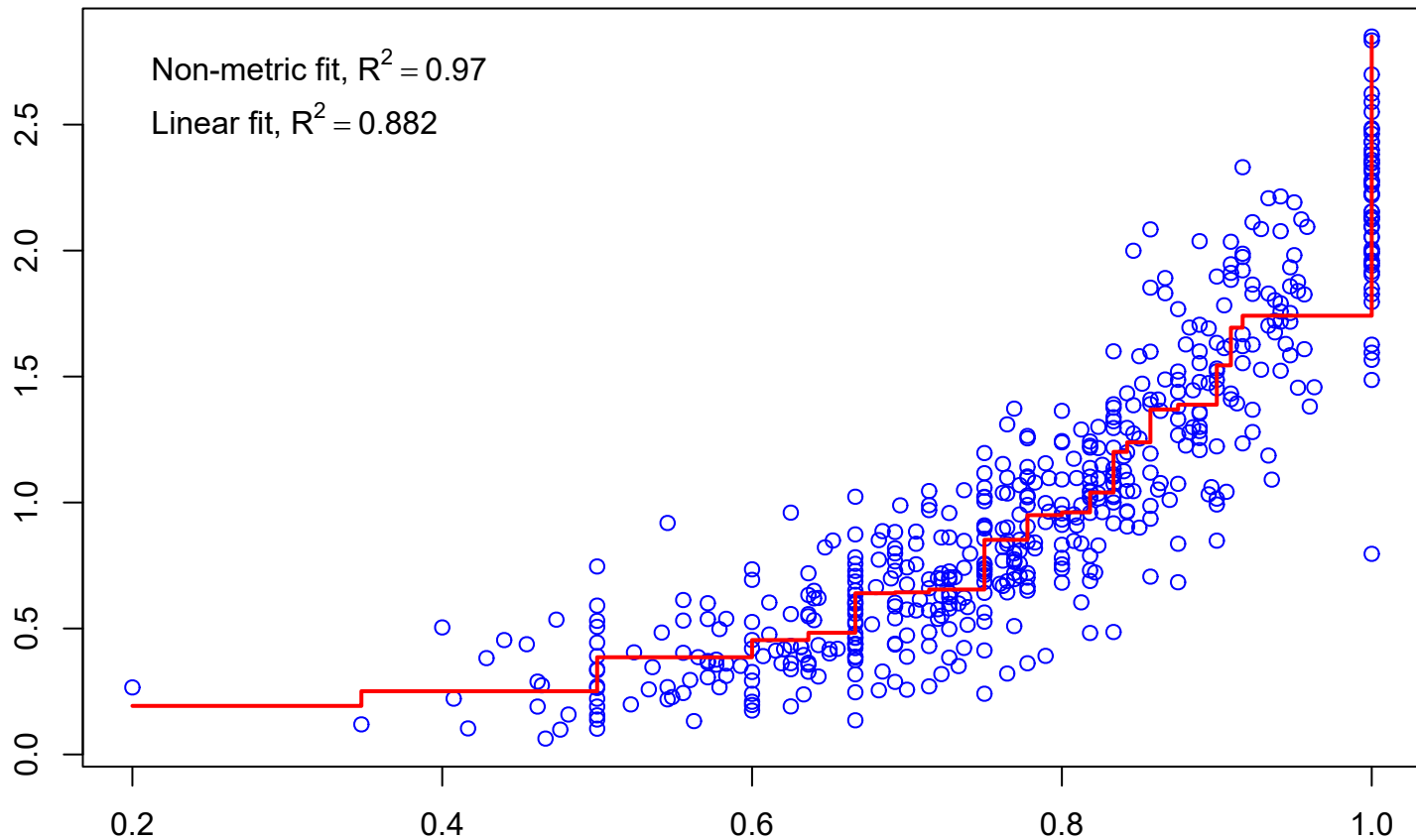

Supplement: Supplemental Information 6 [file peerj-09-11390-s006.pdf]
